# Probiotics and Non-Alcoholic Fatty Liver Disease: Unveiling the Mechanisms of *Lactobacillus plantarum* and *Bifidobacterium bifidum* in Modulating Lipid Metabolism, Inflammation, and Intestinal Barrier Integrity

**DOI:** 10.3390/foods13182992

**Published:** 2024-09-21

**Authors:** Jing Lu, Dilireba Shataer, Huizhen Yan, Xiaoxiao Dong, Minwei Zhang, Yanan Qin, Jie Cui, Liang Wang

**Affiliations:** 1 Xinjiang Key Laboratory of Biological Resources and Genetic Engineering, College of Life Science and Technology, Xinjiang University, Urumqi 830046, China; jinglu@xju.edu.cn (J.L.); dlrb_shataer2021@xju.edu.cn (D.S.); 18536983054@163.com (H.Y.); zhangmw@xju.edu.cn (M.Z.); qingyalan12345@sina.com (Y.Q.); 2 College of Food Science and Light Industry, Nanjing Tech University, Nanjing 211816, China

**Keywords:** probiotics, gut–liver axis, *Lactobacillus plantarum*, *Bifidobacterium bifidum*, structure of intestinal flora, NAFLD mechanism

## Abstract

In recent years, the prevalence of non-alcoholic fatty liver disease (NAFLD) has risen annually, yet due to the intricacies of its pathogenesis and therapeutic challenges, there remains no definitive medication for this condition. This review explores the intricate relationship between the intestinal microbiome and the pathogenesis of NAFLD, emphasizing the substantial roles played by *Lactobacillus plantarum* and *Bifidobacterium bifidum*. These probiotics manipulate lipid synthesis genes and phosphorylated proteins through pathways such as the AMPK/Nrf2, LPS-TLR4-NF-κB, AMPKα/PGC-1α, SREBP-1/FAS, and SREBP-1/ACC signaling pathways to reduce hepatic lipid accumulation and oxidative stress, key components of NAFLD progression. By modifying the intestinal microbial composition and abundance, they combat the overgrowth of harmful bacteria, alleviating the inflammatory response precipitated by dysbiosis and bolstering the intestinal mucosal barrier. Furthermore, they participate in cellular immune regulation, including CD4^+^ T cells and Treg cells, to suppress systemic inflammation. *L. plantarum* and *B. bifidum* also modulate lipid metabolism and immune reactions by adjusting gut metabolites, including propionic and butyric acids, which inhibit liver inflammation and fat deposition. The capacity of probiotics to modulate lipid metabolism, immune responses, and gut microbiota presents an innovative therapeutic strategy. With a global increase in NAFLD prevalence, these insights propose a promising natural method to decelerate disease progression, avert liver damage, and tackle associated metabolic issues, significantly advancing microbiome-focused treatments for NAFLD.

## 1. Introduction

Non-alcoholic fatty liver disease (NAFLD) is a pathological syndrome characterized by excessive fat accumulation in the liver or liver damage, excluding cases caused by alcohol consumption and other specific hepatotoxic factors such as medications, viral infections, and autoimmune conditions [1]. The disease can be classified into several categories based on the presence of inflammation and fibrosis: simple fatty liver, non-alcoholic steatohepatitis (NASH), and NASH-associated cirrhosis [2]. Globally, the prevalence of NAFLD is approximately 25%, with the highest rates found in South America and the Middle East, exceeding 30% [3]. Given its high prevalence, broad impact, and rapidly increasing incidence, studying effective interventions to prevent the progression of NAFLD to NASH is of considerable practical importance.

**Figure 1:**
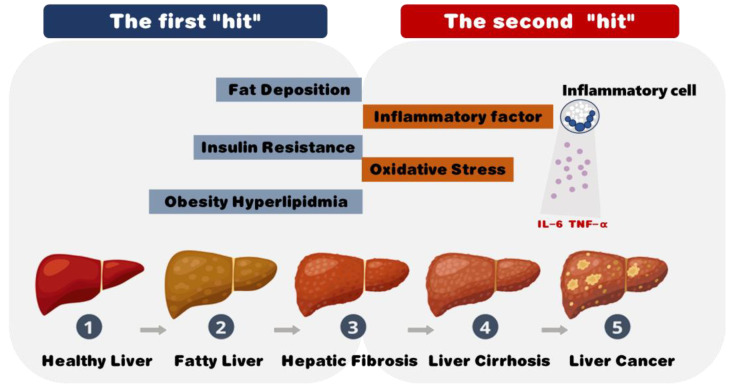
The “Two-hit Hypothesis” of non-alcoholic fatty liver disease. First hit: metabolic disturbances such as obesity, hyperlipidemia, insulin resistance, and fat accumulation lead to liver damage and the development of fatty liver (steatosis); and second hit: inflammatory factors, oxidative stress, and immune cell activation, fueled by cytokines such as IL-6 and TNF-α, exacerbate the damage, advancing the condition to hepatic fibrosis, cirrhosis, and potentially liver cancer. This model underscores the role of metabolic disruptions and inflammation in the progression of NAFLD.

The pathogenesis of NAFLD remains poorly understood, but one of the most widely accepted models is the “Two-Hit Hypothesis” introduced by Alfred Knudson [4]. The first hit involves the accumulation of fatty acids and triglycerides within hepatocytes, leading to simple hepatic steatosis. Subsequently, chronic oxidative stress initiates ongoing damage and inflammation within the mitochondria and hepatocytes, representing the second hit [5]. This second hit enhances the vulnerability of hepatocytes to apoptosis and necrosis, thereby facilitating the development of fibrosis and cirrhosis. Insulin resistance is commonly associated with the second hit (Figure 1). Therefore, insulin resistance, dysregulated fat storage, impaired fat secretion, and oxidative stress are considered the primary pathogenic factors in NAFLD [6].

However, emerging evidence suggests that the high degree of heterogeneity in the pathogenesis and clinical presentations of NAFLD, influenced by various causative factors among patients worldwide [7], indicates that the classic “Two-Hit Hypothesis” may not fully capture the complex and variable nature of NAFLD pathogenesis.

A recent hypothesis emerged to address the shortcomings of the previous two-stage disease progression theory, emphasizing the concurrent and intricate contributions of factors such as insulin resistance, adipokines, bile acids, inflammatory responses, gut microbiota, and other elements in the development of the disease [8,9]. This new perspective particularly highlights the pivotal role of gut microbiota in the progression of NAFLD [5].

Lifestyle modifications for NAFLD primarily involve dietary management and regular physical activity. Studies indicate that engaging in at least 150 min of aerobic exercise per week, spread over 3–5 sessions, can lead to improvements in fatty liver conditions. A diet low in fats and carbohydrates, with minimal sugar and trans fats, and high in fiber, is advised. A 10% reduction in body weight has been shown to significantly enhance liver health in NAFLD patients [10]. A randomized trial involving 154 participants reported a 64% recovery rate with lifestyle and dietary adjustments [11]. In essence, the optimal treatment for NAFLD encompasses a balanced diet, consistent exercise, and efforts to decrease body fat.

Recent research has highlighted the pivotal role of gut microbiota in insulin resistance, obesity, and related metabolic disorders, making the exploration of its connection with NAFLD pathogenesis a rapidly expanding field [12,13,14,15]. The gut–liver axis concept, introduced in 1998, posits a link between gastrointestinal health and liver function [16]. It suggests that intestinal barrier dysfunction enables bacterial translocation, connecting gut integrity to liver health. This allows bacterial endotoxins to reach the liver via the portal vein, activating hepatocytes and leading to the release of inflammatory cytokines, which can cause hepatic immune injury and inflammatory responses. Recognizing the harmful effects of bacterial endotoxins on liver health, there is an increasing focus on identifying microbes that can modulate lipid metabolism and reduce liver inflammation. *Rhodococcus opacus* is particularly notable for its unique lipid metabolism and bioremediation capabilities, making it a compelling subject for further investigation [17]. Nonetheless, the precise mechanisms by which *R. opacus* influences these processes are not fully understood, indicating a significant knowledge gap in the current research. It is important to note that the modulation of NAFLD by gut microbiota presents both potential benefits and risks. 

The beneficial probiotics within the body enhance the integrity of the intestinal mucosal barrier, counteract the proliferation of harmful microorganisms in the intestines, reduce the levels of intestinal endotoxins like lipopolysaccharide (LPS), and restore inflammatory pathways disrupted by excessive gut flora overgrowth, thereby alleviating systemic inflammation [18]. While in vivo and clinical studies have supported the efficacy of probiotics, it is crucial to also consider in vitro and ex vivo research for a comprehensive understanding of their therapeutic potential. Research typically progresses from in vitro studies, through in vivo and ex vivo experiments, to larger clinical trials. Thus, comprehensive investigation across all stages, including in vitro, is essential to fully validate the role of probiotics in NAFLD treatment. 

Probiotics containing *Bifidobacterium* or *Lactobacillus* may offer therapeutic benefits for individuals with NAFLD [19,20,21]. *Bifidobacterium*-based probiotics have been shown to enhance liver function tests (ALT and AST), diminish liver fat content, and lower inflammation and oxidative stress [22,23,24]. *Lactobacillus*-based probiotics, such as those containing *L. rhamnosus*, *L. acidophilus*, and *L. plantarum*, can improve liver health and metabolic parameters by fortifying the gut barrier, modulating metabolism, and reducing inflammatory responses [25,26,27].

These probiotics can be integrated with other treatments, such as dietary modifications, exercise, and medication, to enhance their efficacy against NAFLD. Although NAFLD currently lacks a cure, targeting the gut microbiota with beneficial bacteria like *Bifidobacterium* and *Lactobacillus* presents a promising complementary approach [28]. Ongoing research is continually uncovering their full therapeutic potential in managing NAFLD.

Probiotics have been shown to potentially enhance NAFLD outcomes by regulating gut microbiota, decreasing inflammation and oxidative stress, and improving metabolic function. Nonetheless, further research is required to elucidate the mechanisms by which *Lactobacillus plantarum* and *Bifidobacterium bifidum* interact with NAFLD pathogenesis, the pathways that lead to reduced liver lipid accumulation and oxidative stress, and their roles in immune and lipid metabolism regulation. To this end, this work employs core databases such as PubMed, Web of Science, and ScienceDirect to search for terms related to NAFLD and gut microbiota, including “NAFLD progression”, “probiotic intervention”, and “gut-liver axis”. A total of 539 studies were initially identified through these databases. The inclusion criteria for selecting studies were: (1) original research articles or comprehensive review articles, (2) published in English, and (3) studies released between 2011 and 2024. After the first screening phase, 50 articles that did not provide sufficient methodological details, results, or references were excluded. The remaining 489 studies were further assessed by title and abstract for relevance. Finally, after a full-text evaluation, 102 publications that met all selection criteria were included in this review. We aim to: (1) summarize the increasing prevalence of NAFLD; (2) examine the relationship between the intestinal microbiome and NAFLD pathogenesis, focusing on *Lactobacillus plantarum* and *Bifidobacterium bifidum*; (3) outline how these probiotics affect lipid synthesis and phosphorylation via pathways like AMPK/Nrf2, LPS-TLR4-NF-κB, and SREBP-1, reducing hepatic lipid accumulation and oxidative stress; (4) discuss their role in modifying gut microbiota to inhibit harmful bacteria, alleviate dysbiosis-induced inflammation, and strengthen the intestinal barrier; and (5) highlight their immune regulation and modulation of lipid metabolism to reduce systemic inflammation.

## 2. Intestinal Flora and the Pathogenesis of NAFLD

The gut microbiota plays a pivotal role in the progression of liver diseases, with its structural composition and metabolites exerting influences on NAFLD at various angles and levels.

### 2.1. Structural Composition and Changes in Gut Microbes with NAFLD

The human gastrointestinal tract harbors hundreds of millions of microorganisms, encompassing over 500 different genera and species, predominantly including members of the phyla *Firmicutes*, *Bacteroidetes*, *Proteobacteria*, and *Actinobacteria*. Recent studies increasingly highlighted the close correlation between the occurrence and progression of NAFLD and alterations in the structural composition and relative abundance of the gut flora [29,30,31]. Specifically, *Firmicutes* and *Bacteroidetes* collectively constitute over 90% of the total intestinal microbial population [32]. Research has indicated that the abundance of *Faecalibacterium* and *F. prausnitzii* is reduced in healthy adults and adolescents, whereas *Prevotella*, *Bacteroides*, and *Blautia* exhibit variable changes in abundance, sometimes increasing and other times decreasing [33]. The observed variability in these genera suggests a more complex relationship between gut microbiota and NAFLD pathogenesis than previously thought. The inconsistent patterns of *Prevotella*, *Bacteroides*, and *Blautia* may reflect individual differences in gut flora or represent different stages of disease progression [34]. Microbial shifts may only manifest in advanced stages of NAFLD or in response to dietary and environmental influences [35]. This underscores the significance of accounting for patient-specific factors when interpreting microbiome data in NAFLD research. Moreover, the lack of consistent upregulation of specific genera in NAFLD patients underscores the importance of microbial diversity and resilience, rather than the prevalence of species, in disease progression. These insights suggest that while certain microbial patterns, such as the decreased *Faecalibacterium*, are associated with NAFLD, the overall changes in gut microbiota are highly individualized. This variability necessitates more refined approaches to studying the microbiome’s role in NAFLD and calls for therapeutic strategies that are personalized to individual differences.

The gut–liver axis underscores the link between the gastrointestinal tract and liver, indicating that a compromised intestinal barrier integrity can facilitate bacterial translocation, permitting gut-derived endotoxins to reach the liver via the portal vein [36]. Recent studies indicate that gut dysbiosis may increase intestinal permeability, promoting free fatty acid absorption, bacterial migration, and the release of toxic products like pro-inflammatory cytokines [13,36,37,38,39]. This process activates hepatic cells, such as Kupffer cells, which then secrete inflammatory mediators, contributing to immune damage and inflammatory responses within the liver. Consequently, dysbiosis of the gut microbiota may influence the progression of NAFLD via the gut–liver axis [16].

### 2.2. Gut Microbial Dysbiosis Promotes NAFLD

**Figure 2:**
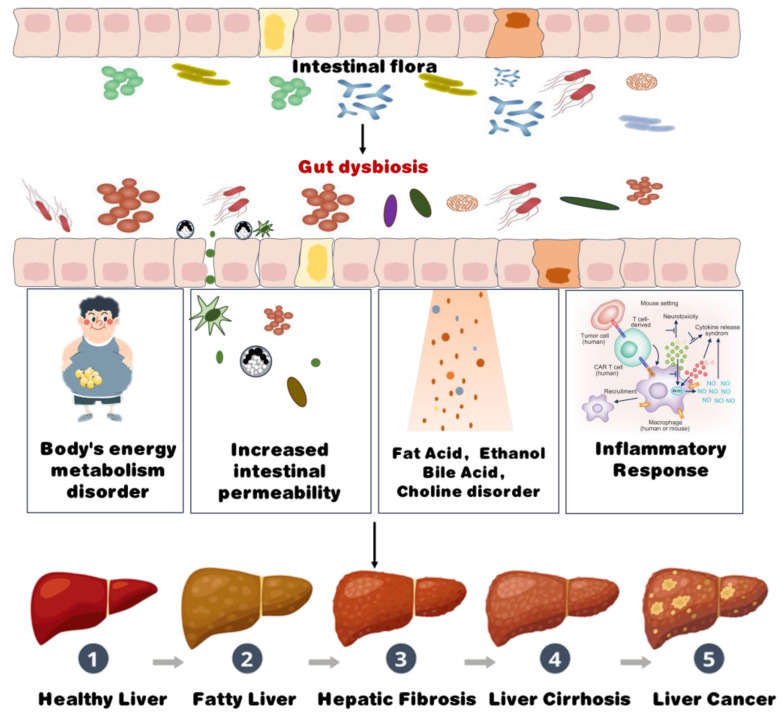
The mechanism of intestinal microbial dysbiosis promoting the occurrence of NAFLD. Intestinal dysbiosis is pivotal in the progression of liver disease, as it disrupts gut flora and initiates a cascade of detrimental effects, including: dysbiosis disrupting energy balance, contributing to obesity and metabolic dysfunction; impaired gut barrier integrity permitting toxins such as lipopolysaccharides to enter the bloodstream, fostering systemic inflammation; dysbiosis disrupting the metabolism of fats, ethanol, bile acids, and choline, further impairing liver function; and microbial byproducts triggering immune activation, leading to chronic liver inflammation.

Modifications in the structural composition and functionality of the gut microbiota can impact the body’s energy absorption and storage processes, precipitating insulin resistance, altering intestinal permeability, and instigating inflammation (Figure 2). Disruptions in microbial homeostasis may also interfere with the synthesis and metabolism of gut flora metabolites, including ethanol, choline, and bile acids [40]. These disruptions can exacerbate hepatic inflammation and potentially accelerate the progression of NAFLD from non-alcoholic simple fatty liver to non-alcoholic steatohepatitis.

#### 2.2.1. Dysbiosis of Intestinal Flora Affects the Body’s Energy Metabolism

The gut microbiota exerts an influence on the host’s energy metabolism and promotes nutrient absorption. Microbial actions can modify the host gene expression by impacting transcription factors such as sterol regulatory element-binding protein-1c (SREBP-1c), carbohydrate response element-binding protein (ChREBP), peroxisome proliferator-activated receptor gamma (PPARγ), and AMP-activated protein kinase (AMPK), leading to triglyceride and fat accumulation while reducing fatty acid oxidative catabolism, thereby regulating the host’s energy metabolism [41]. The composition and relative abundance of certain gut flora also affect energy metabolism and the ectopic deposition of liver fat. The *Firmicutes* and *Bacteroidetes* phyla are primarily responsible for polysaccharide fermentation, and it was observed that a higher proportion of these phyla can absorb more energy from the diet, contributing to fat accumulation in the host [42]. It was reported that there was a decreased diversity in the gut microbial populations and a negative correlation between the presence of *Bifidobacteria* and liver injury in children with NAFLD. 

Furthermore, research showed decreased diversity in gut microbial populations and a negative correlation between *Bifidobacteria* presence and liver injury in children with NAFLD [43]. This underscores the significance of gut microbiota imbalance in the pathogenesis of NAFLD. Therefore, adjusting the composition and abundance of intestinal flora could influence the body’s energy absorption and metabolism, potentially decreasing liver fat accumulation associated with excessive energy intake. This highlights the importance of maintaining a balanced gut microbiota to prevent or mitigate NAFLD development.

#### 2.2.2. Dysbiosis of Intestinal Flora Affects Its Metabolites

The human gut microbiota metabolizes dietary nutrients to produce compounds such as short-chain fatty acids (SCFAs), endogenous ethanol, indoles, and endotoxins, as well as processes bile acids from the liver into secondary bile acids [44]. SCFAs are crucial for maintaining intestinal barrier integrity, modulating immune responses, regulating glucose and lipid metabolism, and influencing appetite control. Consumption of SCFAs can alleviate hepatic steatosis by activating G protein-coupled receptors (GPR)41 and GPR43, which stimulate the release of the gut hormone peptide YY from enteroendocrine cells within the mucosa, ultimately reducing energy intake and mitigating the severity of hepatic steatosis [45].

Ethanol is a significant microbial metabolite, and certain *Enterobacteriaceae* can elevate endogenous ethanol production when the gut flora is disturbed. Upon absorption, ethanol increases intestinal mucosal permeability and reaches the liver via the portal vein, exerting a direct toxic effect. Studies showed that the *Escherichia* species, known to produce ethanol, are more abundant in the intestines of NASH patients, and serum ethanol levels are correspondingly elevated [46]. Furthermore, an increase in the relative abundance of *Aspergillus* and *Prevotella* species were observed in pediatric NAFLD patients with enhanced endogenous ethanol production. Increased endogenous ethanol production in NAFLD and NASH patients underscores the significant link between gut dysbiosis and liver health. Ethanol-induced gut permeability allows harmful substances, such as endotoxins, to reach the liver, aggravating inflammation and fibrosis [47]. Therefore, modulating the gut microbiota, particularly targeting ethanol-producing bacteria, presents a potential therapeutic strategy to manage NAFLD and hinder its progression to more severe conditions like NASH and cirrhosis. This approach could mitigate liver damage by addressing the root cause in the gut. 

Gut microbes play a pivotal role in bile acid transformation and help determine the composition of bile acids. They can inhibit bile acid synthesis by regulating the metabolism of secondary bile acids and by activating nuclear receptors such as the farnesoid X receptor, which suppresses the hepatic enzyme cholesterol 7α-hydroxylase [48]. Elevated bile acid synthesis and an increased primary-to-secondary bile acid ratio in NASH patients were compared to healthy individuals [49]. A positive correlation between taurocholic acid and *Clostridium leptum* was found, along with a negative correlation between *C. leptum* and other bile acids, suggesting *C. leptum* aids in converting primary bile acids to secondary bile acids, potentially reducing liver damage. It is clear that the dysregulation of bile acid synthesis and metabolism is closely linked to metabolic balance in humans, and adjusting bile acid ratios through intestinal flora modulation may offer a new therapeutic approach for managing NAFLD.

Choline is a critical component in hepatic fat metabolism. Its deficiency can lead to NAFLD due to increased fat synthesis and deposition, as well as decreased triglyceride output. Choline in the liver is utilized to produce very low-density lipoprotein (VLDL), and its scarcity impairs VLDL synthesis and secretion, inhibiting fat export and promoting liver fat accumulation, thereby worsening NAFLD [50]. Moreover, choline is a vital phospholipid component of cell membranes, as its deficiency increases intestinal epithelial permeability [51]. The gut microbiota plays a significant role in choline regulation and its anaerobic degradation. *Proteobacteria*, *Firmicutes*, and *Actinobacteria* are the predominant bacteria in the intestine involved in choline metabolism. Research indicates that an increased abundance of γ-proteobacteria is more susceptible to choline deficiency and is more prevalent in NAFLD cases [52]. 

Gut dysbiosis, ethanol production, and choline metabolism are directly linked to the onset and progression of NAFLD. Dysbiosis enhances ethanol generation, impairing the intestinal barrier and permitting endotoxins to penetrate the liver, thereby triggering inflammation and fibrosis that accelerate NAFLD. Concurrently, choline deficiency disrupts fat metabolism, resulting in excessive hepatic fat accumulation. Diminished choline metabolism regulation by gut bacteria further aggravates NAFLD. Collectively, these factors highlight the critical role of the gut microbiota, ethanol production, and choline metabolism in NAFLD development.

#### 2.2.3. Dysbiosis of Intestinal Flora Causes an Inflammatory Response in the Body

The intestinal microbiota can induce chronic systemic inflammation through various pathways. Research indicates that the continuous lysis of Gram-negative bacteria in the gut releases LPS, which binds to its receptor CD14 and then the TLR4 receptor on immune cells, leading to the acceleration of inflammasome production and pro-inflammatory cytokines, thereby initiating the inflammatory cascade involving the NF-κB pathway [53]. The activation of this inflammatory pathway results in the release of substantial amounts of tumor necrosis factor-alpha (TNF-α), which contributes to lipid peroxidation and fibrosis in hepatocytes, thereby promoting the progression of fatty liver disease [54]. 

Intestinal homeostasis relies on the balanced interplay between pro-inflammatory TH17 cells and anti-inflammatory TReg cells. Probiotics like *Lactobacillus casei* and *Bifidobacterium longum* were shown to stimulate IL-10 production by CD4^+^ T cells, mediating protective effects against intestinal diseases by modulating the TReg cell response, indicating that intestinal probiotics can influence the host immune system [55]. Disruptions in the bacterial microbiota can result in dysregulated adaptive immune cells, potentially contributing to conditions such as inflammatory bowel disease.

An imbalance in the intestinal flora can exacerbate the progression of NAFLD, whereas beneficial probiotics can mitigate this process by modulating the immune response. *Faecalibacterium prausnitzii* stimulates the proliferation of antigen-specific T cells and inhibits the production of the pro-inflammatory cytokine IL-8, while promoting the secretion of the anti-inflammatory cytokine IL-10 in peripheral blood mononuclear cells, thus reducing the production of pro-inflammatory factor interferon-gamma (IFN-γ) [56]. Additionally, a 15 kDa anti-inflammatory protein was identified in the metabolites of *F. prausnitzii*, which was shown to effectively counteract inflammation by inhibiting the NF-κB signaling pathway [57]. 

In summary, intestinal dysbiosis triggers chronic inflammation by upsetting the equilibrium between pathogenic and beneficial bacteria, leading to the release of LPS from Gram-negative bacteria that activates the NF-κB pathway. This leads to increased production of TNF-α production, causing lipid peroxidation and fibrosis in the liver, thereby advancing NAFLD. Probiotics like *Lactobacillus*, *Bifidobacterium*, and *Faecalibacterium prausnitzii* help restore microbial balance, reduce inflammation, and modulate immune responses. By enhancing gut barrier integrity and fostering beneficial bacteria, probiotics support gut health and counteract the progression of NAFLD.

#### 2.2.4. Dysbiosis of Intestinal Flora Increases Intestinal Permeability

The interaction between epithelial cells and the intestinal microbiota leads to the formation of a protective barrier consisting of a layer of mucus-secreting IgA molecules, immune cells, microcolonies of specialized bacteria, enzymes, and host-microbial metabolites [58]. This barrier prevents harmful microorganisms and their metabolites from reaching specific receptors on living cells. However, dysbiosis disrupts this barrier, enhancing mucosal permeability and exposing intestinal mucosa and the liver to potentially pro-inflammatory bacterial products. Evidence suggests that dysbiosis-induced increased permeability raises levels of gut-derived TLR ligands in the portal vein, activating Kuffer cells and stellate cells in the liver and stimulating pro-inflammatory and pro-fibrotic pathways via cytokines such as IL-1, IL-6, and TNF-α [59]. 

Recent studies indicate that probiotic interventions can preserve barrier integrity and alleviate intestinal hyperpermeability associated with NAFLD [60,61]. Briskey et al. observed that administering a probiotic mixture (comprising *Bifidobacterium*, *Lactobacillus*, and *Streptococcus*) to obese mice fed a high-fat diet significantly lowered serum glucose, cholesterol, and triglyceride levels and improved intestinal barrier permeability by upregulating tight junction proteins ZO-1 and ZO-2 at the molecular level [62]. 

Additionally, *Lactobacilli* were reported to inhibit NF-κB activation by gut flora and reduce hepatic inflammation through AMPK activation and SIRT1 expression. Furthermore, *Lactobacilli* elevate the expression of tight junction proteins Occludin and ZO-1 by phosphorylating AMPK and AKT proteins, maintaining the intestinal mucosal barrier function and modulating the intestinal microbial structure [63]. Thus, probiotic intervention that modifies intestinal tissue tight junction proteins, decreases intestinal permeability, and reduces inflammation is a crucial mechanism for slowing NAFLD progression.

These findings suggest that targeted probiotic therapies could be used to prevent or slow NAFLD progression by strengthening the intestinal barrier and reducing inflammation. These therapies may serve as supplements to existing treatments or as standalone interventions for those at high risk of NAFLD due to diet or genetic predisposition. However, several limitations must be noted. The current studies are primarily based on animal models, which may not fully capture the complexity of human NAFLD. Additionally, the most effective probiotic strains, dosages, and combinations are still unclear, and the long-term effects on gut microbiota and liver function are not well understood. Future research should focus on conducting large-scale, randomized, controlled trials in humans to validate these findings, determine optimal probiotic formulations, and further investigate the underlying mechanisms.

## 3. Evidence of Probiotics in NAFLD

**Figure 3:**
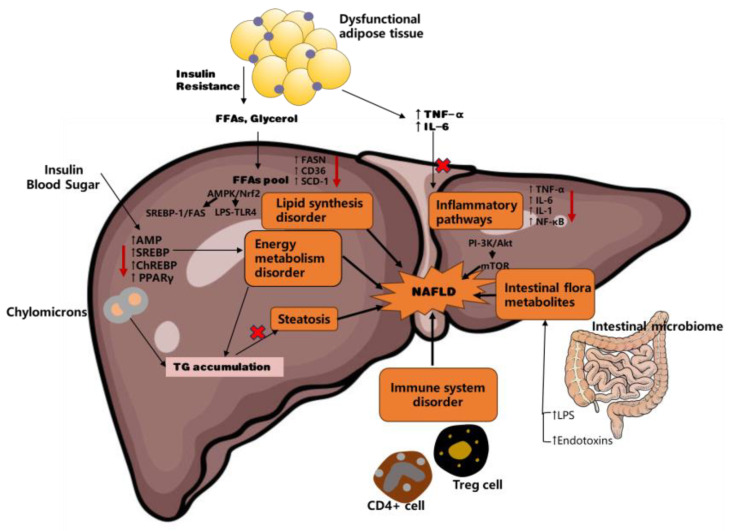
Possible mechanisms of probiotics in the treatment of NAFLD. Insulin resistance, dysfunctional adipose tissue, lipid metabolism disorders, inflammatory signaling, and intestinal dysbiosis are intertwined in the progression of liver disease. Adipose tissue insulin resistance leads to increased release of free fatty acids (FFAs) and glycerol, which accumulate in the liver, impairing lipid metabolism. Elevated levels of inflammatory cytokines, such as TNF-α and IL-6, foster chronic inflammation. Upregulation of fatty acid synthase (FASN), cluster of differentiation 36 (CD36), and stearoyl-CoA desaturase-1 (SCD-1) drive excessive fatty acid synthesis, resulting in steatosis and lipid synthesis disorders. Signaling molecules like AMPK/Nrf2, SREBP-1 and carbohydrate-responsive element-binding protein (ChREBP) exacerbate metabolic imbalance, further increasing triglyceride (TG) accumulation. Liver inflammatory pathways activated by cytokines TNF-α, IL-6, IL-1, and NF-κB cause immune dysfunction, as evidenced by CD4^+^ T cells and regulatory T cells (Tregs). Gut dysbiosis, marked by increased lipopolysaccharides (LPS) and endotoxins, exacerbates liver inflammation. Elevated insulin and blood sugar levels enhance de novo lipogenesis (DNL) through peroxisome proliferator-activated receptor gamma (PPARγ), SREBP-1, and ChREBP, further promoting TG accumulation. Red arrows denote intervention and regulation strategies. Thick black arrows indicate direct regulatory factors, while thin black arrows represent indirect regulatory factors. Short thin black arrows signify molecular-level regulatory factors.

Probiotics, as a natural component of the human body, are introduced to enhance the colonization advantage within the intestinal tract, fostering the development of a balanced intestinal microflora and ecosystem. They also suppress competition with pathogenic bacteria, thereby reversing harm or injury caused by detrimental bacteria. Based on the relationship between the intestinal microbiota and the pathogenesis of NAFLD, the benefits of probiotics in treating NAFLD can be primarily summarized as follows: (1) Enhancing the intestinal mucosal barrier function and decreasing intestinal epithelial permeability. (2) Altering the structural composition of the intestinal microbiota by countering the growth of harmful microbes and competitively inhibiting their pathogenicity. (3) Lowering intestinal endotoxin levels, such as LPS. (4) Repairing the inflammatory pathway triggered by excessive intestinal flora growth and reducing systemic inflammation through cellular immunomodulation, including CD4^+^ T-cells and TReg cells (Figure 3).

Currently, common probiotics are classified into three primary groups: *Lactobacillus* species, *Bifidobacterium* species, and *Streptococcus* species. Recent research has increasingly focused on the potential utility of *Lactobacillus* and *Bifidobacterium* species as supplementary treatments for NAFLD, suggesting promising therapeutic applications.

### 3.1. Application of Lactobacillus in the Treatment of NAFLD

The *Lactobacillius* species include *Lactobacillus acidophilus*, *Lacticaseibacillus casei*, *Lactobacillus plantarum*, *Lactobacillus rhamnosus*, among others, with *L. plantarum* being the most extensively studied in relation to NAFLD. *L. plantarum* is widely utilized in food and industrial fermentation, and its safety has been acknowledged by the European Food Safety Authority and the U.S. Food and Drug Administration [64]. Numerous studies have elucidated the therapeutic potential of *L. plantarum* in mitigating NAFLD, with comprehensive data presented in Table 1.

Research has demonstrated that fatty acid synthesis in the liver is regulated by both insulin and glucose, with membrane-bound transcription factors such as SREBP-1c and ChREBP, along with PPARγ and AMPK, playing crucial roles in NAFLD pathogenesis through their involvement in the independent regulation of adipogenesis-related enzyme transcription and activity [74]. *L. plantarum* WW improved dysregulated hepatic lipid metabolism, reduced oxidative stress, and alleviated NAFLD by activating the PPAR signaling pathway and participating in lipid metabolism when administered to rats on a high-fat diet [66]. Additional studies reported that *L. plantarum* DSM20174, *L. plantarum* ZJUIDS14, and *L. plantarum* Q16 enhanced lipid metabolism and relieved hepatic steatosis due to insulin resistance by downregulating PPARγ expression [67,73,75]. Moreover, *L. plantarum* ATCC14917 and *L. plantarum* FZU3013 were found to lower serum total cholesterol (TC) and triglyceride (TG) levels and reduce lipid accumulation in the liver by modulating the glycerophospholipid metabolic pathway [72].

AMPK, a heterotrimeric protein functioning as a cellular energy sensor, is activated in response to elevated AMP levels indicative of low energy states. Activation of AMPK promotes ATP generation, such as through the oxidation of fatty acids, while also inhibiting ATP-consuming processes through phosphorylating regulatory proteins or influencing gene expression within these pathways. Research revealed that *L. plantarum* NA136 modulates fatty acid metabolism and protects against oxidative stress via the AMPK/Nrf2 pathway, simultaneously suppressing the SREBP-1c/FAS signaling pathway to inhibit adipogenesis from preadipocytes [68,69]. Additionally, *L. plantarum* Q16 was shown to enhance hepatic energy metabolism by engaging the FGF21/adiponectin/AMPK/PGC-1α signaling axis [73]. Similarly, *L. plantarum* MG5289 impacts energy metabolism and reduces excessive lipid accumulation in the liver by modulating AMPK proteins [71].

Recent observations of NAFLD implicate oxidative stress as a key factor in its early pathogenesis, suggesting that it plays a pivotal role in disease progression. This oxidative stress arises predominantly from increased oxidative reactions, including the oxidation and peroxidation of mitochondrial fatty acids and the ω-oxidation of ultra-long-chain fatty acids. The depletion of effective antioxidants leads to an accumulation of reactive oxygen species, exacerbating NAFLD [76]. Notably, *L. plantarum* has been shown to potentially delay the progression from NAFLD to NASH by mitigating oxidative stress. Li et al. demonstrated that an *L. plantarum* NCU116 treatment of high-fat diet-induced NAFLD in rats restored liver function within 5 weeks, concurrently reducing oxidative stress markers and hepatic fat deposition [70]. Additionally, *L. plantarum* NCU116 significantly lowered endotoxin levels and pro-inflammatory cytokines and modulated the microbial flora composition. These findings suggest that *L. plantarum* NCU116 exerts its beneficial effects on NAFLD through dual mechanisms: downregulating genes linked to lipogenesis and upregulating those associated with lipolysis and fatty acid oxidation [75]. Park et al. (2020) further reported that *L. plantarum* ATG-K2 and *L. plantarum* ATG-K6 enhanced antioxidant activity in vivo by regulating the expression of antioxidant enzymes like superoxide dismutase (SOD), glutathione peroxidase (GPx), and catalase (CAT), primarily via the Nrf2/Keap1 signaling pathway, thereby reducing hepatic lipid accumulation [66]. 

The incidence and progression of NAFLD are closely linked to alterations in the gut microbiota’s structure and abundance. *L. plantarum* NA136 enhances intestinal barrier integrity by fostering the proliferation of beneficial bacteria such as *Allobaculum*, *Lactobacillus*, and *Bifidobacterium*, indicating that *L. plantarum* NA136 may alleviate NAFLD primarily through the modulation of the gut microbiota and the gut–liver axis [69]. Using 16S rRNA gene sequencing, *L. plantarum* DSM20174 modifies the diversity of the murine gut microbiota and adjusts specific bacterial taxa at the family, genus, and species levels, leading to decreased lipid accumulation and inflammatory mediators [67].

In conclusion, *L. plantarum* effectively corrects dysregulated hepatic lipid metabolism and suppresses excessive hepatic lipid accumulation, primarily through pathways associated with lipid metabolism, including PPAR, LPS-TLR4-NF-κB, AMPK/Nrf2, and SREBP-1/FAS signaling. It enhances in vivo antioxidant capacity and reduces oxidative stress by modulating the expression of antioxidant enzymes and genes related to adipogenesis and fatty acid β-oxidation, such as SOD, GPx, and CAT, as well as carnitine palmitoyltransferase 1-α [77]. Additionally, *L. plantarum* influences the structural composition and abundance of the gut microbiota, promoting the growth of beneficial commensals like *Allobaculum* and *Lactobacillus*, thereby enhancing the intestinal barrier integrity and exerting its effects through the gut–liver axis [16]. By enhancing the composition of the gut microbiota and strengthening the gut–liver axis, *Lactobacillus plantarum* not only treats NAFLD but also addresses its root causes. This probiotic presents a promising holistic strategy for the long-term management of NAFLD and merits further clinical research for broader application.

### 3.2. Application of Bifidobacteria in the Treatment of NAFLD

*Bifidobacterium bifidum*, a common member of the human intestinal microbiota, facilitates the digestion, breakdown, and metabolism of fats. It also curtails the proliferation of pathogens like *Staphylococcus* in the intestine and enhances intestinal motility, thereby improving the digestive tract’s functionality in individuals with fatty liver conditions [78]. Accumulating clinical evidence underscores the critical role of *Bifidobacteria* in the progression of NAFLD. The application of *Bifidobacteria* to modulate the gut microbiota offers a novel and secure approach for the management of NAFLD.

#### 3.2.1. Clinical Research

Recently, intestinal dysbiosis and altered bacterial metabolites significantly contribute to the progression of NAFLD to hepatocellular carcinoma (HCC) in murine models [79]. In patients with NAFLD-HCC, unique microbial and metabolomic profiles were identified, characterized by a notable decline in *Bifidobacteria* and an increase in *Bacteroides* and *Ruminalococcaceae* [79]. These shifts in gut microbiome composition are associated with the progression from NAFLD to hepatocellular carcinoma, mediated by changes in gut–liver axis interactions and microbial metabolites. As the disease severity escalates, there is a notable depletion of probiotics, including *Bifidobacterium* and *Lactobacillus*. To delineate the roles of these probiotics, Valerio Nobili conducted a study analyzing *Bifidobacteria* and *Lactobacillus* in stool samples from healthy children and those with NAFLD using 16S RNA sequencing of the intestinal microbiome [80]. Their findings provided insights into the metabolic pathways and microbial communities associated with NAFLD, highlighting the potential importance of these probiotics in disease pathogenesis. Sequencing results were predominantly *B. longum*, *B. bifidum*, *Bifidobacterium adolescentum*, *Lactobacillus zeae*, *Lactobacillus vaginalis*, *Lactobacillus shortum*, *Lactobacillus rumenus*, and *Lactobacillus mucosae*. In particular, *L. mucosae* was significantly higher in NAFLD (*p* = 0.01313) and NASH (*p* = 0.01079) compared to the healthy group. In addition, *Bifidobacterium* spp. were more abundant in the healthy group. This suggested that *Bifidobacterium* spp. were more protective for NAFLD patients. More interestingly, the authors emphasized that *Bifidobacterium* and *Lactobacillus* strains may have different effects on NAFLD.

Clinical trials have yielded preliminary evidence supporting the efficacy of orally administered *Bifidobacteria* in treating NAFLD. A three-year study explored the therapeutic impact of *B. longum* supplementation in NAFLD patients between January 2003 and June 2006, enrolling a total of 66 subjects who were randomized to receive *B. longum* along with fructose and lifestyle modifications (diet and exercise) or lifestyle modifications alone. Assessments were conducted at weeks 0, 6, 12, 18, and 24, evaluating parameters such as aspartate aminotransferase (AST), alanine aminotransferase, bilirubin, albumin, total cholesterol, HDL and low-density lipoprotein (LDL) cholesterol, triglycerides, fasting glucose, insulin, c-peptide, c-reactive protein (CRP), and TNF-α, among others. The outcomes indicated that *B. longum* supplementation significantly decreased TNF-α, CRP, serum AST levels, HOMA-IR, serum endotoxin, steatosis, and NASH activity scores compared to the group receiving only lifestyle modifications. This suggests that long-term intake of *B. longum* might be a viable therapeutic option for managing NAFLD [23].

As research delves deeper into NAFLD, interventions targeting the gut–liver axis have emerged as a focal point. The impact of probiotics on NAFLD focused on small intestinal microbes, permeability, and inflammatory markers within the intestinal epithelium [81]. Following a six-month regimen of a multi-strain probiotic blend containing *Lactobacillus* and *Bifidobacterium*, no substantial alterations were observed in blood biochemistry; however, there was a significant reduction in inflammatory cytokines such as IFN-γ and TNF-α. A 16S rRNA sequencing of small intestinal biopsy specimens revealed an uptick in *Actinobacteria* and a downturn in *Proteobacteria* in the probiotic-treated cohort. Given that *Proteobacteria* are often implicated in inflammation-driven diseases [82]. 

Current research on probiotics for NAFLD predominantly focuses on short-term interventions. For instance, a 12-week randomized controlled trial by Ma et al. demonstrated that supplementation with *Bifidobacterium* and *Lactobacillus* significantly decreased liver fat and inflammatory markers, but the limited duration precluded the evaluation of long-term effects and safety [83]. Similarly, an 8-week study by Eslamparast et al. showed improvements in serum ALT, AST levels, and lipid profiles but also lacked long-term data [84].

These results imply that targeted probiotic therapies, especially those involving *Bifidobacteria*, could be beneficial in managing NAFLD by lowering inflammation and improving liver function, potentially serving as adjuncts to existing treatments, particularly for patients who do not respond to lifestyle modifications. Nonetheless, further research is required to ascertain the efficacy of various probiotic strains, dosages, and treatment durations.

The majority of studies are based on small clinical trials that may not fully capture the complexity of human NAFLD, and the emphasis on particular probiotic strains might not produce consistent outcomes across various patient groups. Moreover, despite the documentation of microbial changes, their long-term clinical benefits are not yet clear. Future research should concentrate on conducting large-scale, randomized controlled trials to validate these results, identify the most effective probiotic formulations, and elucidate the underlying mechanisms.

#### 3.2.2. Research on Rodent NAFLD Models 

The primary mechanism of *Bifidobacteria* in treating NAFLD can be partly explained through the modulation of the AMPK/SREBP-1c and PPAR signaling pathways, as detailed in Table 2. *Bifidobacteria* suppress the transcription of several lipid-synthesis-related enzymes by regulating hepatic sterol regulatory element-binding proteins, thereby curtailing fatty acid synthesis at the molecular level and leading to decreased levels of TG, TC, and LDL [85,86]. For instance, *B. bifidum* BGN4 and *B. longum* BORI notably hindered the elevation of liver fat in hyperlipidemic mice, demonstrating *Bifidobacteria*’s inhibitory impact on lipid accumulation [87]. Similarly, *Bifidobacterium animalis* subsp. *lactis* MG741, *B. longum* LC67, *Bifidobacterium lactis* V9, and *B. longum* DD98 diminished the synthesis of hepatic fatty acids, ameliorating hepatocyte ballooning and hepatic steatosis by lowering hepatic TC and TG levels [88,89,90]. Furthermore, *B. longum* MG723 and *B. longum* MG731 rectify lipid and glucose metabolic disturbances in NAFLD by modulating genes linked to fatty acid and cholesterol synthesis, including Dhcr7, Dhcr24, Sc5d, Hsd17b12, and FASN [91]. 

Two pivotal mechanisms of *B. bifidum* in the treatment of NAFLD involve the inhibition of inflammatory cytokine release and the reduction in tissue inflammation, alongside the maintenance of intestinal barrier integrity. *B. longum* MG723 and *B. longum* MG731 have been shown to diminish the production of pro-inflammatory markers such as F4/80, MCP-1, TGF-β, IL-1β, and IL-18 [91]. Additionally, *Bifidobacterium animalis* subsp. *lactis* MG741 elevates the expression of tight junction proteins like ZO-1 and occludin, while suppressing inflammatory mediators, thereby alleviating intestinal inflammation and preserving the integrity of the intestinal barrier [88]. *Bifidobacterium* supplementation significantly lessened hepatic fat accumulation (0.10 ± 0.03 g/g liver tissue compared to 0.16 ± 0.03 g/g in the model group), they did not observe improvements in intestinal permeability, suggesting a need for further evidence regarding *Bifidobacteria*’s impact on the gut barrier [93]. *B. longum* R0175 reduces oxidative damage, inflammation, and hepatocyte apoptosis by activating the Nrf2 pathway, concomitant with a decrease in pro-inflammatory cytokines like IL-1, TNF-α, and chemokines [97]. Moreover, *B. lactis* V9 and *B. longum* DD98 abate the production of pro-inflammatory chemokines (IL-6, IL-1β, and TNF-α), retarding NAFLD progression [89]. *B. bifidum* modulates inflammatory responses and immune cell populations, lowering pro-inflammatory cytokine levels and the Th17 cell ratio, thereby mitigating liver inflammation [96]. 

The third key mechanism of *B. bifidum* in the management of NAFLD involves the regulation of intestinal dysbiosis, the reduction in pathogenic bacteria, and the alteration of the gut microbial composition. Intestinal dysbiosis exacerbates NAFLD, and *B. longum* R0175 has been shown to alleviate dysbiosis by augmenting the relative abundance of beneficial bacteria (like *Alloprevotella* spp.) and diminishing the relative abundance of potentially detrimental species (such as *Acetatifactor muris*, *Butyricimonas* spp., and *Oscillibacter* spp.), thereby mitigating liver injury and fibrosis [92]. *B. longum* LC67 modulates intestinal dysbiosis by reducing *Firmicutes* and *Proteobacteria*, which are often implicated in inflammation-driven diseases [95]. By enhancing the relative abundance of *Faecalibaculum* and *Lactobacillus* while reducing that of *Tyzzerella*, *Escherichia-Shigella*, *Nestiinimonas*, *Osillibacter*, and *Ruminiclostridium*, *B. bifidum* indirectly influences lipid metabolism pathways, inhibiting the initial synthesis of fatty acids. These alterations in the microbial community and its metabolites contribute to the anti-inflammatory and hepatoprotective effects of probiotics.

The fourth principal mechanism of *Bifidobacteria* in treating NAFLD pertains to the modulation of intestinal flora metabolites, influencing tissue inflammation, de novo fatty acid synthesis, and intestinal barrier integrity. *B. longum* MG723 and *B. longum* MG731 elevate the expression of bile acid synthesis genes such as Cyp71 and Cyp27a1, affecting bile acid metabolism and energy metabolism [91]. *B. bifidum* reduces the relative abundance of *Tyzzerella*, *Escherichia-Shigella*, *Nestiinimonas*, and *Osillibacter* while increasing the relative abundance of *Faecalibaculum* and *Ruminiclostridium*, leading to higher levels of propionic acid and butyric acid. These metabolites mediate NAFLD by modulating lipid metabolism and intestinal permeability, thereby curbing hepatic inflammation and fat deposition [95]. Additionally, *Bifidobacteria* restore short-chain fatty acids and tryptophan metabolite imbalances by modulating immune response-linked gene pathways, thereby sustaining immune equilibrium and alleviating NAFLD progression [94]. 3.2.3. Research on Chicken NAFLD Models.

Previous research indicated that *Bifidobacterium* significantly contributes to alleviating metabolic disease [98,99]. Recent research also demonstrated the potential of *Bifidobacterium* in alleviating NAFLD symptoms in chicken models. Dev K et al. demonstrated that using *B. bifidum* and mannan oligosaccharides (MOS) as a synbiotic in broiler chicken feed, at 0.2% MOS and 10^−6^ CFU/g *B. bifidum*, led to significant improvements in growth performance, feed efficiency, gut health, and antioxidant levels (*p* < 0.01). This synbiotic combination reduced the presence of harmful bacteria (e.g., *Escherichia coli* and *Clostridium perfringens*) and increased beneficial bacteria (e.g., *Lactobacillus* and *Bifidobacterium*). It also elevated serum antioxidant enzymes and HDL cholesterol levels while reducing blood glucose, TG, TC, and atherosclerosis-related markers (*p* < 0.01) [100]. In chicken models, *B. bifidum* strengthens the intestinal barrier, reducing endotoxin translocation to the liver, thereby mitigating inflammation and fibrosis associated with NAFLD. By enhancing gut health and modulating the microbiota, *B. bifidum* effectively decreases liver inflammation and fat accumulation. Its ability to regulate the gut–liver axis shows potential for addressing metabolic disorders in poultry, improving both intestinal integrity and liver function, making it a promising approach for managing NAFLD in chickens.

Thus, this paper has explored the protective effects and underlying mechanisms of *Bifidobacterium* on NAFLD, focusing on lipid metabolism, intestinal flora equilibrium, inflammatory response, and intestinal barrier integrity, offering fresh perspectives and strategies for NAFLD prevention and therapy.

## 4. Limitations of the Studies

Previous research has underscored the advantages of probiotics in managing NAFLD, with studies indicating that strains such as *Lactobacillus* and *Bifidobacterium* can enhance gut health and decrease systemic inflammation [37,101]. For example, probiotic supplementation led to significant reductions in liver fat and improvements in lipid profiles among NAFLD patients [102]. However, while earlier studies frequently concentrated on isolated pathways, this review emphasizes the intricate mechanisms through which *L. plantarum* and *B. bifidum* operate, engaging multiple signaling pathways and immune responses. This comprehensive perspective aligns with the complexity of NAFLD pathogenesis and suggests that targeting multiple pathways concurrently could represent a more effective therapeutic approach. These insights could guide the creation of innovative probiotic therapies as either complementary or alternative treatments for NAFLD.

## 5. Conclusions and Perspectives

This review examined the interplay between the intestinal microbiome and NAFLD pathogenesis, highlighting the significant contributions of *L. plantarum* and *B. bifidum*. These probiotics modulate lipid synthesis genes and phosphorylated proteins via pathways like the AMPK/Nrf2, LPS-TLR4-NF-κB, AMPKα/PGC-1α, SREBP-1/FAS, and SREBP-1/ACC signaling axes to diminish hepatic lipid accumulation and oxidative stress during NAFLD progression. By altering the intestinal microbial structure and abundance, they counteract the proliferation of harmful bacteria, ameliorating the inflammatory cascade triggered by dysbiosis and fortifying the intestinal mucosal barrier. They also engage in cellular immunoregulation, such as CD4^+^ T cells and Treg cells, to quell systemic inflammation. Additionally, both *L. plantarum* and *B. bifidum* influence lipid metabolism and immune responses by modulating gut metabolites, including propionic and butyric acids, thereby inhibiting hepatic inflammation and fat accretion. 

In the future, it is essential to conduct further studies not only to identify the most effective integrative therapies but also to deepen our understanding of the microbiota composition. A more thorough comprehension of the microbial community in relation to liver disease progression could aid in delineating the stages of liver disease and serve as a valuable diagnostic tool. Expanding research in this area could also provide insights into how probiotics such as *L. plantarum* and *B. bifidum* could be leveraged to tailor therapeutic strategies, optimizing the management of NAFLD and potentially offering personalized probiotic interventions for disease prevention and treatment.

## Figures and Tables

**Table 1:** Palliative effects of *Lactobacillus plantarum* on NAFLD.

Lactobacillus Plantarum Strain	Source of Strains	Research Objects	Mechanism Pathways	Reported Functions	Reference
*L. plantarum* WW	Naturally Fermented Soybean Paste (College of Food Science, Shenyang Agricultural University, Shenyang, China)	Male C57BL/6 Mice	PPAR signaling pathway Up-regulation of PPAR expression	Improvement of lipid metabolism disorders Reduced oxidative stress	Cao et al. [65]
*L. plantarum* ATG-K2 *L. plantarum* ATG-K6	Kimchi, a Korean Traditional Fermented Cabbage (AtoGen Co Ltd., Daejeon, South Korea)	Male Wistar Rat	Nrf2/Keap1 signaling pathway Modulation of SREBP-1 and ACC signaling Regulation of the expression of antioxidant enzymes and lipogenesis-related genes (SOD, GPx and CAT)	Reduced lipid accumulation in the liver Enhanced antioxidant enzyme activity Reduced AST and ALT levels	Park et al. [66]
*L. plantarum* DSM20174 (involved ingesting L.p (IAM 12477)	Pickled Cabbage (Spanish Type Culture Collection, Valencia, Spain)	Male C57BL/6 Mice	PPAR signaling pathway Regulating adipogenic genes FASN Regulation of fatty acid β-oxidation genes CPT1 LPS-TLR4-NF-κB signaling pathway	Improved glucose and lipid homeostasis Reduction in white fat inflammation Changed relative abundance of bacteria	Riezu-Boj et al. [67]
*L. plantarum* NA136 *L. plantarum* NA136	Chinese Traditional-style Pickles (China Center for Type Culture Collection, Wuhan, China)	Male C57BL/6J Mice Male C57BL/6J Mice	AMPK/Nrf2 signaling pathway SREBP-1/FAS signaling pathway	Reduced adipose tissue mass, lipid levels, AST and ALT levels Improvement of intestinal barrier integrity Promoted the growth of probiotics such as *Allobaculum*, *Lactobacillus*, and *Bifidobacteria*	Zhao et al., Zhao et al. [68,69]
*L. plantarum* NCU116	Pickled Vegetables (State Key Laboratory of Food Science and Technology, Nanchang University, Nanchang, China)	Male Sprague Dawley Rats	Regulating adipogenic genes FASN Regulation of fatty acid β-oxidation genes CPT1	Reduced endotoxin and pro-inflammatory cytokines Reduce oxidative stress Reduced lipid accumulation in the liver Regulation of colon bacterial flora and hepatic lipid metabolism expression	Li et al. [70]
*L. plantarum* ZJUIDS14	6-Month-OldnBreastfed Babies Feces (State Key Laboratory of Food Science and Technology, NanChang, China)	Male C57BL/6 Mice	Up-regulation of PPAR expression Activate AMPK protein	Improved mitochondrial function Reduced insulin resistance	Cao et al. [65]
*L. plantarum* MG5289	Food origin (MEDIOGEN Co., Ltd., Jechon, Republic of Korea)	Male C57BL/6 Mice	Activate AMPK protein	Reduced pro-inflammatory factor TNF-α, IL-1β and IL–6 Reduced lipid accumulation in the liver Reduced AST and ALT levels	Lee et al. [71]
*L. plantarum* ATCC14917	Pickled Cabbage (China General Microbiological Culture Collection Center, CGMCC, Beijing, China)	Male Sprague Dawley Rats	LPS-TLR4-NF-κB signaling pathway	Reduced serum total cholesterol (TC), triglycerides (TG) Improve intestinal flora imbalance	Wen et al. [72]
*L. plantarum* FZU3013	Traditional Brewing Process of Hongqu Rice Wine (College of Food Science, Fujian Agriculture and Forestry University, Fujian, China)	Male Kunming Mice	Glycerophospholipid metabolic pathway Fatty acid degradation pathway	Reduced abnormal levels of serum TG, TC and LDL-C Reduced lipid accumulation in the liver	Wen et al. [72]
*L. plantarum* Q16	Yogurt (The College of Food Science and Technology of Nanjing Agricultural University, Nanjing, China)	Male specific pathogen-free (SPF) Mice	AMPKα/PGC-1α signaling pathway Up-regulation of PPAR expression Reduced expression of FAS, ACC, SCD-1, Srebp-1c and ATGL	Reduced lipid accumulation in the liver Improve intestinal flora imbalance Inhibited the growth of endotoxin-producing microorganisms	Chao et al. [73]

**Table 2:** Palliative effects of *Bifidobacterium* on NAFLD.

Bifidobacterium Strain	Source of Strains	Research Objects	Mechanism Pathways	Reported Functions	Reference
*B. longum* BORI *B. bifidum* BGN4	The intestines of Korean infants. (Bifido Co., Ltd., Hongchun, Republic of Korea)	ICR Mice	Modulation of cellular inflammatory factors; Fatty acid biosynthetic pathways;	Reduced hepatic TG, TC, and LDL-C Reduced serum levels of AST, ALT, TG Reduced pro-inflammatory factor TNF-α, IL-1β Improvement of hepatocyte hydropathy and hepatic steatosis Reduced lipid accumulation in the liver	Li et al. [87]
*B. longum* MG723 *B. longum* MG731	Human Sources (Biomedical Science Institute, Gangnam Severance Hospital, Yonsei)	Korean Individuals C57BL/6N Mice and Germ-Free Mice	Modulation of cellular inflammatory factors; Fatty acid biosynthetic pathway: CYP7A1 and CYP27A1; Bile acid synthesis;	Reduced body weight gain Down regulated inflammation, lipid synthesis, glucose synthesis upregulated bile acid metabolism and energy metabolism Promoted oxidative phosphorylation in adipose tissue	Kim et al. [91]
*B. longum* R0175	Human Gastrointestinal Tract (Lallemand., Inc., Quebec, Canada)	Male Sprague Dawley (SD) Rats	Modulation of cellular inflammatory factors	Reduced the level of liver damage Reduced serum levels of AST and total bile acids (TBAs) Reversal of intestinal dysbiosis in liver-injured rats Increasing the relative abundance of potentially beneficial bacteria (e.g., *Alloprevotella* spp.) and decreasing the relative abundance of potentially harmful bacteria (e.g., *Acetatifactor muris*, *Butyricimonas* spp. and *Oscillibacter* spp.)	Wang et al. [92]
*B. longum* R0175	Human Gastrointestinal (Lallemand., Inc., Quebec, Canada) Tract Lallemand, Inc. (France)	Male C57BL/6J Mice	Nrf2/Keap1 signaling pathway	Reduced oxidative stress Reduced inflammatory response Amelioration of apap-induced microbiota dysbiosis to attenuate liver injury	Li et al. [87]
*B. longum* LC67	Human Gastrointestinal Tract (Biomedical Science Institute, Gangnam Severance Hospital, Yonsei)	Korean Individuals C57BL/6N Mice and Germ-Free Mice	AMPK/NK-κB signaling pathway Maintenance of intestinal barrier function integrity protein: ZO-1, Occludin	Reduced liver and blood levels of AST, ALT, TG, and LPS Regulation of intestinal bacterial flora disorders: reduced the number of *Firmicutes* and *Proteobacteria* in the intestinal flora	Kim et al. [91]
*B. Longum* CGMCC 2107	Human Gastrointestinal Tract (Institute of Bio-medicine, Shanghai Jiao Tong University, Shanghai, China)	Male Sprague Dawley Rats	No validation at the molecular level	Reduced lipid accumulation in the liver Reduced serum levels of AST, ALT, TG No improvement in intestinal permeability	Xu et al. [93]
*B. breve CKDB002 * *B. longum CKDB004*	Newborns Feces (Chong Kun Dang bioCorp, Seoul, South Korea)	32 Patients with NAFLD and 25 Healthy Subjects Male C57BL/6J Mice	β-oxidation	Restoration of short-chain fatty acids and tryptophan metabolite disorders Reduced lipid accumulation in the liver	Yoon et al. [94]
*B. animalis* ssp. *lactis* MG741	Human Infant Feces (Mediogen Co., Ltd., Jecheon, Chungbuk, Republic of Korea.)	Male C57BL/6J Mice	Modulation of SREBP-1 and ACC signaling SREBP-1/FAS signaling pathway Maintenance of intestinal barrier function integrity protein: ZO-1,Occludin	Reduced lipid accumulation in the liver Regulation of serum metabolic disorders	Do et al. [88]
*B. lactis* V9	Healthy Mongolian Children Feces (The Key Laboratory of Dairy Biotechnology and Bioengineering, Educational Ministry of China, Mongolia, China)	Male Wistar Rats	Modulation of SREBP-1 and ACC signaling AMPK signaling pathway PPAR signaling pathway LPS-TLR4-NF-κB signaling pathway	Reduced liver and serum levels of AST, ALT, TG, and LPS Reduced the level of liver damage Reduced pro-inflammatory factor TNF-α, IL-1β, and IL-6	Yan et al. [89]
*B. bifidum* BGN4	Korean infants Feces (China General Microorganisms Culture Collection Center, Institute of Microbiology, Beijing, China)	Male Sprague Dawley Rats	SREBP-1/FAS signaling pathway PPAR signaling pathway	Reduced hepatic TG, TC, and LDL-C Reduced serum levels of AST, ALT	Ren et al. [86]
*B. adolescentis* BA3, BA5, Z25	Human Feces (The Food Biotechnology Centre of Jiangnan University, Wuxi, China)	Male C57BL/6J mice	Regulating the structure and abundance of intestinal flora Lipid metabolism pathway	Modulation of intestinal flora, increasing the relative abundance of *Faecalibaculum* and *Lactobacillus*, decreasing the relative abundance of *Tyzzerella*, *Escherichia-Shigella*, *Nestiinimonas*, *Osillibacter* and *Ruminiclostridium;* Increased propionic and butyric acid levels, regulated lipid metabolism and intestinal permeability, ultimately inhibiting liver inflammation and fat accumulation	Wang et al. [95]
*B. Bifidum* BGN4	Korean infants Feces (The BeNa Culture Collection, Suzhou, China)	Male C57BL/6J Mice	Modulation of cellular inflammatory factors AMPK signaling pathway Cellular immune homeostasis: T helper cells LPS-TLR4-NF-κB signaling pathway	Reduced insulin resistance Reduced lipid accumulation in the liver Decreased pro-inflammatory factor levels and Th17 cells’ ratio, increased Treg cells’ ratio Reduced insulin resistance Reduced hepatic TG, TC, and LDL-C Reduced serum levels of AST, ALT, TG Reduced pro-inflammatory factor TNF-α, IL-1β, and IL-6	Nian et al. [96]

## Data Availability

No new data were created or analyzed in this study. Data sharing is not applicable to this article.
